# Early white matter pathology in the fornix of the limbic system in Huntington disease

**DOI:** 10.1007/s00401-021-02362-8

**Published:** 2021-08-26

**Authors:** Sanaz Gabery, Jing Eugene Kwa, Rachel Y. Cheong, Barbara Baldo, Costanza Ferrari Bardile, Brendan Tan, Catriona McLean, Nellie Georgiou-Karistianis, Govinda R. Poudel, Glenda Halliday, Mahmoud A. Pouladi, Åsa Petersén

**Affiliations:** 1 Translational Neuroendocrine Research Unit, Department of Experimental Medical Science, Lund University, BMC D11, 22184 Lund, Sweden; 2 Translational Laboratory in Genetic Medicine (TLGM), Agency for Science, Technology and Research (A*STAR), Singapore, 138648 Singapore; 3 Department of Medical Genetics, British Columbia Children’s Hospital Research Institute, University of British Columbia, Vancouver, V5Z 4H4 Canada; 4 School of Psychological Sciences, Monash University, Clayton, VIC 3180 Australia; 5 Department of Pathology, Alfred Hospital, Melbourne, VIC Australia; 6 The Brain and Mind Centre and Faculty of Medicine and Health, School of Medical Sciences, University of Sydney, Sydney, Australia; 7 Department of Physiology, National University of Singapore (NUS), Singapore, 117597 Singapore; 8 Present Address: Evotec SE, HD Research and Translational Sciences, 22419 Hamburg, Germany

**Keywords:** Huntingtin, Oligodendrocyte, Fornix, MRI, RNA sequencing

## Abstract

Huntington disease (HD) is a fatal neurodegenerative disorder caused by an expanded CAG repeat in the huntingtin (*HTT*) gene. The typical motor symptoms have been associated with basal ganglia pathology. However, psychiatric and cognitive symptoms often precede the motor component and may be due to changes in the limbic system. Recent work has indicated pathology in the hypothalamus in HD but other parts of the limbic system have not been extensively studied. Emerging evidence suggests that changes in HD also include white matter pathology. Here we investigated if the main white matter tract of the limbic system, the fornix, is affected in HD. We demonstrate that the fornix is 34% smaller already in prodromal HD and 41% smaller in manifest HD compared to controls using volumetric analyses of MRI of the IMAGE-HD study. In post-mortem fornix tissue from HD cases, we confirm the smaller fornix volume in HD which is accompanied by signs of myelin breakdown and reduced levels of the transcription factor myelin regulating factor but detect no loss of oligodendrocytes. Further analyses using RNA-sequencing demonstrate downregulation of oligodendrocyte identity markers in the fornix of HD cases. Analysis of differentially expressed genes based on transcription-factor/target-gene interactions also revealed enrichment for binding sites of SUZ12 and EZH2, components of the Polycomb Repressive Complex 2, as well as RE1 Regulation Transcription Factor. Taken together, our data show that there is early white matter pathology of the fornix in the limbic system in HD likely due to a combination of reduction in oligodendrocyte genes and myelin break down.

**Supplementary Information:**

The online version contains supplementary material available at 10.1007/s00401-021-02362-8.

## Introduction

Huntington disease (HD) is a fatal autosomal dominant disorder caused by an expanded CAG repeat in the huntingtin (*HTT*) gene. There are no disease-modifying treatments available. Clinical diagnosis is based on the manifestation of typical motor signs in combination with a genetic test. Motor symptoms are associated with neuronal loss in the striatum of the basal ganglia and the cerebral cortex. Psychiatric and cognitive symptoms often precede the motor component of HD by many years but less is known about the underlying biological mechanisms. The hypothalamus and the limbic system have been suggested to play a role for non-motor symptoms and signs of HD [6]. The hypothalamus has been shown to be affected more than a decade prior to the onset of clinical disease [1, 2], with pathological changes in the hypothalamus associated with the early non-motor features of HD [25, 26]. Other parts of the limbic system have been less studied in HD.

Emerging studies have indicated pathology also occurs in white matter (WM) in HD, with MRI-based imaging studies in HD gene carriers showing early and progressive changes in large WM tracts using group-wise comparisons [4, 40, 46, 52, 62]. Furthermore, animal models of HD show that WM and myelination abnormalities are an early disease feature appearing before the manifestation of behaviour abnormalities or neuronal loss [14, 24, 53] and that treating oligodendrocyte abnormalities can alleviate the neurological manifestations of HD in mice [14, 21]. Preclinical studies have shown that expression of mutant HTT (mHTT) in oligodendrocytes leads to transcriptional downregulation of myelin regulating factor (MRF), a master transcription factor controlling myelin genes [24]. Most studies have focused on the large WM tract corpus callosum. Post-mortem evaluation of oligodendrocytes in clinical HD is limited to striatal changes and have shown increased oligodendrocyte density [37], possibly reflecting attempts to remyelinate [3], despite a reduction in postnatally generated oligodendrocytes [13]. These studies are consistent with an early preclinical oligodendroglial abnormality in HD that impacts on the subsequent neurodegenerative disease processes and their clinical expression.

The largest WM tract in the hypothalamus is the fornix. The fornix connects the hippocampus to the hypothalamus and the mamillary bodies as part of Papez circuit in the limbic system [31, 55]. Here, we use a variety of methods to determine if the fornix in the limbic system is affected and to what extent the changes involve WM and oligodendroglia pathology in HD.

## Material and methods

### Study design

In the present study, we investigated the presence and nature of changes in the fornix in the limbic system in HD using three sets of clinical material. We assessed the volume of the fornix using MRI from a 3 T scanner in the IMAGE-HD cohort study, a multimodal neuroimaging study including clinical, neurocognitive and neuropsychiatric assessments of 31 pre-HD gene carriers, 32 symptomatic HD (symp-HD) patients, and 30 healthy controls (Table 1) [7, 42]. We assessed the volume of the fornix and the number of oligodendrocyte-like profiles using stereology and ultrastructural changes of myelin under the electron microscope in fixed post-mortem human tissue from 9 HD and 8 control cases (Table 2). We measured myelin breakdown, markers of axonal damage and the transcription factor MRF using Western Blot and performed RNA sequencing of frozen human fornix tissue from five HD cases and four control cases (Table 2).

**Table 1:** Demographic, clinical, neurocognitive and neuropsychiatric data of the Image-HD study participants

	Control	Pre-HD	Symp-HD
*n*	30	31	32
Gender (F/M)	21/9	19/12	13/19
Age (years)	44 ± 14	41 ± 9	53 ± 9*
CAG repeat length		42 ± 2	43 ± 3
UHDRS-M		0.91 ± 1.26	18.50 ± 10.79
Estimated years to onset		15 ± 6	
CAP score		81 ± 10	107 ± 11
Disease burden score		274 ± 49	376 ± 73
Symptom duration (years)			2 ± 2
Verbal IQ	118 ± 10	116 ± 11	115 ± 11
SDMT	55 ± 10	52 ± 9	36 ± 12*^,#^
STROOP	110 ± 18	105 ± 18	84 ± 23*^,#^
SCOPI—total OCD	80 ± 20	82 ± 25	91 ± 25
FrSBe—total score	86 ± 27	90 ± 23	92 ± 23
HADS: A	5 ± 3	6 ± 3	6 ± 3
HADS: D	2 ± 3	2 ± 3	3 ± 2
BDI II	3 ± 3	8 ± 10	8 ± 7*

Data is presented as mean ± SD. The data were analyzed by one-factor ANOVA followed by post-hoc tests when appropriate

*UHDRS-M* Unified Huntington’s Disease Rating Scale—motor subscale score (Pre-HD, UHDRS < 5; Symp-HD, UHDRS ≥ 5), *CAG* cytosine-adenine-guanine (number of repeats > 40 is full penetrance), *CAP* CAG age product, *SDMT* Symbol Digit Modalities Test, *STROOP* STROOP speeded word reading task (number of correct words), *FrSBe* Frontal Systems Behaviour Scale, *SCOPI* Schedule of Compulsions Obsessions and Pathological Impulses, *HADS A* Hospital Anxiety and Depression Scale—Anxiety Sub Score, *HADS D* Hospital Anxiety and Depression Scale—Depression Sub Score, *BDI II* Beck Depression Inventory score Version II

*p < 0.05 compared to controls

^,#^*p* < 0.05 compared to pre-HD

**Table 2:** Demographic data for post-mortem analyses

Case	Age/sex	PMD	SD	Grade	ABC score	Brain weight	RIN
HD1*	59/m	40	16	2	A1 B0 C2	na	
HD2*	63/f	6	9	2	A0 B0 C0	1030	
HD3*^,^^	51/m	33	13	2	A0 B0 C0	1154	
HD4*	57/m	43	10	2	A0 B0 C0	1100	
HD5*^,^^	57/m	28	6	3	A0 B0 C0	1400	
HD6*	32/m	42	13	4	A0 B0 C0	na	
HD7*	37/f	10	10	4	A0 B0 C0	1030	
HD8*^,^,#,§^	71/m	41	12	2	A2 B0 C0	1270	6.8
HD9*^,^,#,§^	69/f	2	20	2	A0 B0 C0	1149	8.2
HD10^#,§^	68/m	10	13	3	A0 B0 C0	1184	8.4
HD11^#,§^	57/f	22	22	4		800	6.9
HD12^#^	61/m	17	17	4		1280	
HD13^,§^	39/m	10	13	4		1047	7.6
C1*^,^^	64/f	5			A1 B0 C0	1262	
C2*^,^^	68/m	11			A0 B0 C0	1380	
C3*^,^^	75/m	24			A1 B2 C0	1500	
C4*^,^^	79/m	8			A0 B1 C0	1242	
C5*	38/m	14			A0 B0 C0	1620	
C6*	63/f	12			A0 B0 C0	1416	
C7*	44/m	15			A0 B0 C0	1448	
C8*	55/m	39			A0 B0 C0	1560	
C9^#,§^	69/m	24				1290	7.2
C10^#,§^	57/m	48				1372	7.2
C11^#,§^	73/m	43				1532	6.7
C12^#,§^	67/f	26				1298	7.4

Data are indicated as mean ± SD

*RIN* RNA integrity number, *na* not available

*Cases used for stereological analyses

^^^Cases used for electron microscopy

^#^Cases that were used for Western Blot

^§^Cases used for RNA sequencing. Age is indicated in years. Postmortem delay (PMD) is indicated in h. SD (symptom duration) is indicated in years. Brain weight is indicated in g. Grade refers to Vonsattel grade for neuropathological classification of HD [56]. ABC score was obtained by ranking along three parameters (amyloid, Braak, CERAD) according to current consensus criteria [27, 48]

### Analyses of white matter tracts in 3 T MRI

The volumetric measurements of WM tracts were performed on MR images from the IMAGE-HD study (Table 1) [7, 42]. Pre-HD and symp-HD participants underwent genetic testing and had a CAG repeat length ranging from 39 to 50. All participants were clinically assessed using the Unified Huntington's Disease Rating Scale (UHDRS) motor subscale. HD participants with a UHDRS motor score ≤ 5 were included in the pre-HD group and those with UHDRS motor score > 5 were included in the symp-HD group. Estimated years-to-onset of diagnostic motor symptoms were calculated for the pre-HD participants using the formula established by Langbehn and colleagues, accounting for CAG repeat length and current age [29]. Healthy controls were matched for age, gender and IQ (National Adult Reading Test 2nd edition, NART-2) [38] to the pre-HD individuals. The MRI images were obtained on a Siemens 3 Tesla scanner. T1-weighted images were acquired for each participant using the following acquisition sequence parameters: 192 slices, 0.9 mm slice thickness, 0.8 mm × 0.8 mm in-plane resolution, TE = 2.59 ms, TR = 1900 ms, flip angle = 9°. The total intracranial volume (ICV) was calculated as described previously [18]. Processing and the manual measurements of the images after acquisition were performed with the ANALYZE 10.0 software package (Biomedical Imaging Resource, Mayo foundation, Rochester, MN) with a digitized pen and drawing pad.

The volumetric measurements of the WM tracts were derived on T1-weighted images, which were preprocessed by acquiring cubic spine interpolation and a resizing of voxels to 0.42 × 0.42 × 0.45 mm. The original slices were reformatted into the coronal plane. Freesurfer automatic segmentation (Freesurfer 3.0), as previously described in [15], was used to segment the corpus callosum. The volume of the column of the fornix was measured within the hypothalamic region, from bregma 2.7 mm to bregma 9.3 mm (based on the Atlas of the Human Brain by Mai et al.) [32]. The same component of the column of the fornix within the hypothalamic region was assessed in sections from MRI and human post-mortem tissue. To ensure validity, we evaluated with intraclass correlation coefficient (ICC) for two independent raters that measured the fornix volume on a subset of randomly selected 15 cases. The difference in the estimation of the fornix volume between the two raters was 1.29 ± 3% (mean ± SEM) and ICC = 0.970, indicating reproducibility of this method in 3 T MRI. A single rater then estimated the fornix volume bilaterally on all cases using between 15 and 20 sequential slices from a rostral to caudal direction. The rater was blinded to the identify of all cases.

### Post-mortem human material

Following approvals from three Australian Brain Banks (PID073, PID0111, PID167), serial formalin-fixed (*N* = 17 cases; 9 HD and 8 controls) and fresh-frozen (dry ice then stored at − 80 °C, *N* = 9 cases; 5 HD and 4 controls) coronal tissue blocks of the entire human hypothalamus, including the fornix, were obtained from the Sydney Brain Bank at Neuroscience Research Australia, the New South Wales (NSW) Brain Tissue Resource Center at the University of Sydney, and the Victorian Brain Bank. Study inclusion was based on the dominant type of diagnostic neuropathology present that was screened by the NSW Brain Banks, being either no significant neuropathology (incidental, non‐diagnostic pathologies allowed) using current consensus criteria [27, 48] or HD pathology [56]. Demographic data are shown in Table 2. All participants had given their informed consent prior to the donation of their brains for research purposes through regional brain donor programs approved by the institutional Human Research Ethics Committees associated with these tissue collection research programs.

### Histological and stereological analyses of the fornix

The formalin fixed coronal tissue blocks of the human hypothalamus were cryoprotected in 30% sucrose and serially cut at a thickness of 50 μm on a freezing microtome to form 15 series of equally spaced sections throughout the entire antero-posterior extent of the hypothalamus. Sections were mounted on glass slides and one series of sections stained for Nissl substance [0.5% cresyl violet (CV), ICN Biomedicals Inc, stabilized with 10% acetic acid] and myelin [0.1% luxol fast blue (LFB), Solvent Blue 38, Sigma, stabilized with lithium carbonate]. The volume of the column of the fornix within the hypothalamic region was determined on blind-coded serial myelin stained sections using the Computer Assisted Toolbox Software (New CAST) module in VIS software (Visiopharm, Horsholm, Denmark) and applying the optical fractionator using the Cavalieri method [59]. The borders of the column of fornix were outlined in each serial section between bregma 2.7 to 9.3 mm (based on the Atlas of the Human Brain by Mai et al. 2008) [32]. The cross-sectional area of the fornix was then computed, and the volume determined by multiplying the cross-sectional areas by the distance between sections (750 µm). Estimation of the total number of oligodendrocyte-like profiles was obtained by stereological analyses of CV/myelin-stained sections. Nissl-stained cells were determined to be oligodendrocyte-like profiles using the same criteria as in Myers et al. [37] and as described in detail in Garcia-Cabezas et al. [19]: small cells with rounded, dark nuclei without visible cytoplasm. This method was chosen as the possibility of reliable immunohistochemistry of oligodendrocyte markers is limited in post-mortem human tissue by prolonged fixation besides issues with specificity and sensitivity of available antibodies [19]. The total numbers of oligodendrocyte-like profiles were obtained employing the optical fractionator principle [59] using the following formula: *N* = Σ*Q* × 1/fraction × number of series, where *N* is equal to the total number of neurons, Σ*Q* is the number of oligodendrocyte-like profiles counted per brain, the fraction is the percentage of total volume that was used for sampling, and the number of series was 15.

### Analyses using electron microscopy

For electron microscopy (EM), a 2-mm punch using a disposable biopsy punch (Miltex) of the fornix was collected from 50 μm-thick free-floating formalin sections of 4 HD and 4 controls (indicated in Table 2). The tissue punches were then rinsed in 0.1 M Sorensen’s phosphate buffer solution followed by post-fixation for 1 h in 1% osmium tetroxide. After incubation, sections were rinsed once more in 0.1 M Sorensen’s phosphate buffer and then dehydrated in increasing concentration of acetone. The tissue punches were then flat embedded in polybed epoxy resin. Once hardened, the blocks were trimmed and sectioned at 50 nm using an ultramicrotome (Leica EMU C7). Sections were then contrasted in 4% uranylacetate at 40 °C for 20 min, followed by rinsing in distilled H_2_O. Finally, sections were stained in 0.5% lead citrate for 2 min at room temperature. EM images were obtained using a FEI Tecnai at 120 kV. Axon and myelin fiber diameters were measured using ImageJ (NIH). 125 axons were measured per case. The integrity of myelin rings of the fornix was evaluated on 46 randomly selected EM images from 4 HD and 4 controls. For each image the total number of complete intact myelin rings as well as misshapen myelin rings were determined. Myelin rings in which myelin sheets contained swellings, disruptions, folds and irregular shapes were classified as misshapen.

### Collection of human fornix tissue for molecular analyses

Prior to all tissue handling, all equipment and tools were cleaned with RNaseZap (Ambion). The frozen hypothalamic tissue was fixed in frozen state in a coronal position using OCT Cryomount (Histolab) and serially sectioned from a rostral to caudal direction at − 15 °C on a Cryostat (Microm HM 560). The tissue was cut at a thickness of 200 μm and collected on pre-cooled glass slides and stored at − 20 °C overnight before being micro dissected. Every 1 mm, a 20-μm section was collected and stained for Nissl substance (0.5% CV, ICN Biomedicals Inc, stabilized with 10% acetic acid) for orientation purposes during the dissection. The CV-stained sections were assessed under a light microscope and then scanned and printed on A4 paper. The anatomical position of the fornix in the scanned images was determined using the atlas of Mai et al. [32] as reference for the dissections. WM tissue from the fornix was collected with 3 mm disposable biopsy punches (Miltex) and stored in RNA-free tubes that were immediately frozen on dry ice and kept at − 80 °C until processed for RNA isolation.

### Western blots

Tissue collected from the fornix was used for analyses using the MBP-QD9 antibody, which has been shown to detect fragmented myelin [34, 35, 58] and to measure protein levels of MRF using Western Blot. The samples were lysed in 1:10 weight/volume with 1% SDS lysis buffer supplemented with protease and phosphatase inhibitors (Roche) and sonicated for 15 s at 40 Hz. The samples were then incubated for 10 min on ice followed by centrifugation at 14,000 rpm for 10 min. Protein concentrations was measured using the DC Protein Assay Kit (Bio-Rad) according to the manufacturer’s recommendation. The protein lysates were boiled at 95 degrees for 10 min in Laemmli Loading Buffer (Bio-Rad). Either 30 or 15 mg of lysate was loaded into each lane on a 4–15% or a 10–20% TGX (Bio-Rad Criterion TGX Precast gels) and run for 30 min at 90 V, followed by 1 h at 120 V (until the loading dye had crossed the bottom end of the gels). Samples were then transferred to a PVDF membrane by using the Trans-blot Turbo Transfer System (Bio-Rad). Membranes were then blocked for 1 h at room temperature (RT) in 5% skim-milk in Tris-buffered saline buffer + 0.1% Tween 20 (Sigma) (TBS-T). The membranes were then incubated at 4 °C with primary antibodies in 5% skim-milk in TBS-T. The following primary antibodies were used: 1:1000 Neurofilament (mab5262, made in mouse, Millipore), 1:1000 MBP-QD9 (mab8817, made in mouse, Abnova), and 1: 500 MRF (abn45, made in rabbit, Millipore). After three washes of 10 min in TBS-T, the membranes were incubated for 1 h at room temperature with 1:10,000 peroxidase-labeled secondary antibody goat anti-mouse (Santa Cruz) or goat anti-rabbit (Santa Cruz) diluted in 5% skim-milk in TBS-T. Membranes were then washed three times for 10 min in TBS-T and developed using the Clarity Western ECL substrate kit (Bio-Rad). Finally, membranes were imaged using Bio-Rad ChemiDoc MP imaging system. The densitometry analysis of the bands was performed using the computerized image analysis tool Image Lab version 2.0.1.

### RNA sequencing

Total RNA was isolated from tissue samples using RNeasy Lipid Tissue Kit (Qiagen) with an on-column DNase digestion (RNase-free DNase set, Qiagen) according to supplier’s recommendations. RNA quantity was measured on a NanoDrop 2000 spectrophotometer (Thermo Scientific). RNA integrity number (RIN), an indicator for appropriate preservation of RNA integrity, was used to assess the RNA quality of the human postmortem tissue [10, 16, 51] in SCIBLU Genomics, Affymetrix unit at Lund University using Agilent 2100 Bioanalyzer. RNA integrity was determined for all samples before proceeding with the analyses (Table 2).

For RNA sequencing, subsequent library preparation using TruSeq Stranded mRNA LT Sample Prep Kit and paired-end 150-bp sequencing and 25 million reads/per sample were performed by Macrogen (Singapore). We performed RNA sequencing quantification directly from the reads with Salmon v0.9.1 [41] using GRCh37 (hg19; Ensembl release 75) human reference genome. The data were imported into R using tximport v1.11.7 [50] to obtain gene-level counts, filtered for genes with a minimum CPM of 1 in all samples, and subsequently analyzed for differentially expressed genes with edgeR v3.3.0 [44]. Genes were considered statistically differentially expressed if the false discovery rate (FDR) was < 10%. Gene ontology analysis was performed with clusterProfiler v3.16.0. PCA and representative plots were derived from log CPM values with a prior count of 1. The list of glial markers was derived from human single-cell RNA-sequencing data (PanglaoDB, Table 1, online resource) [17]. Transcription factor/epigenetic mediator enrichment analysis was performed on differentially expressed genes using EnrichR [28].

### Statistical analysis

Data are shown as mean ± SD, unless otherwise specified. GraphPad Prism7 and PASW19 statistical package (SPSS) was used for statistical analyses. Significance was considered for *p* < 0.05. After verifying normal distributions using Kolmogorov–Smirnov tests, one-way ANOVA followed by a Tukey’s post hoc test or Kruskal–Wallis followed by Dunn’s post hoc test were performed. For correlation analyses, Spearmans’ *ρ* tests were performed.

## Results

### MRI and post-mortem tissue cohorts of HD and control cases

The cohort of analyzed MRI from the IMAGE-HD study included data from 31 pre-HD gene carriers, 32 symp-HD patients, and 30 healthy controls with clinical, neurocognitive and neuropsychiatric assessments [22]. Healthy controls were matched for age, gender and IQ to the pre-HD individuals. Symp-HD patients were significantly different to pre-HD and the control groups in age and results of the tests SDMT, and STROOP (Table 1). In the cohort of nine HD cases and eight control cases with fixed postmortem tissue, there were no differences in age (HD: 55 ± 13 years; C: 61 ± 14 years), sex (HD: 33% females; C: 25% females) or post-mortem delay (HD: 27 ± 17 h; C: 16 ± 11 h) between the groups but an expected significant reduction in brain weight in the HD group (1162 ± 134 g) compared to the control group (1429 ± 133 g) (Students *t* test *p* < 0.001) (Table 2). ABC score, obtained by ranking along three parameters (amyloid-β plaques, Braak neurofibrillary tangles and CERAD neuritic plaques) according to current consensus criteria [27, 48], was overall low but appeared higher in the control group. Similarly, in the cohort of five HD cases and four control cases with frozen postmortem tissue, there were no differences in age (HD: 61 ± 13 years; C: 67 ± 7 years), sex (HD: 40% females: C: 25% females), post-mortem delay (HD: 17 ± 15 h; C: 35 ± 12 h) or RIN (HD: 7.6 ± 0.7; C: 7.1 ± 0.3) between the groups but an expected significant reduction in brain weight in the HD group (1090 ± 181 g) compared to the control group (1373 ± 112 g) (Students *t* test, *p* < 0.05) (Table 2).

### Smaller fornix volume already in prodromal HD in the IMAGE-HD study

**Fig. 1:**
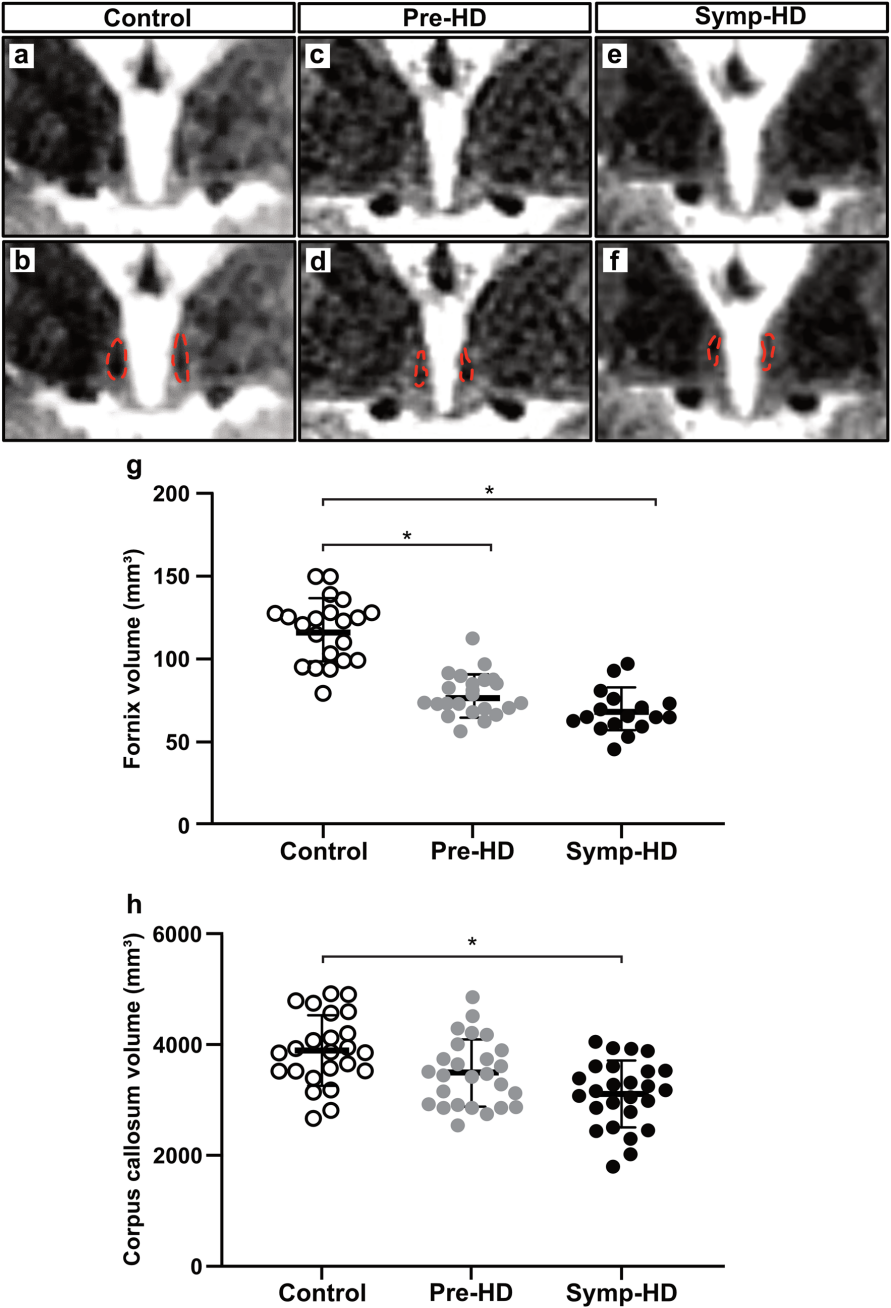
Smaller white matter tract volumes in prodromal and symptomatic HD patients in the IMAGE-HD study. Overview of the hypothalamic region containing the column of the fornix in coronal T1-weighted MRI acquired at 3 T together with the corresponding image which includes dashed red lines to illustrate how the fornix region was delineated from a control (**a**, **b**), pre-HD (**c**, **d**) and symp-HD (**e**, **f**). The fornix volume was significantly smaller in both the pre-HD and symp-HD groups compared to control groups (**g**) (one-way ANOVA, followed by Tukey’s post hoc tests *p < 0. 0.0001). At the same timepoint, the segmented volume of corpus callosum was smaller in symp-HD compared to control group (h) (one-way ANOVA, followed by Tukey’s post hoc tests *p < 0. 0.05), but no significant difference was detected between pre-HD and control group (*p* = n.s.). Data are expressed as mean ± SD

We began by investigating whether the fornix would be affected in prodromal and manifest HD in MRI from the IMAGE-HD cohort, and confirm atrophy of the corpus callosum [9, 45]. We have previously performed volumetric analyses of the hypothalamus in this cohort, in which we defined robust anatomical landmarks to outline the hypothalamic region [18]. The total volume of the hypothalamus was not different in pre-HD or symp-HD compared to controls [18]. Using the same hypothalamic landmarks as before, we outlined the column of the fornix within the hypothalamic region on T1-weighted MRI obtained on a 3 T scanner (Fig. 1a–f). We estimated the volume of the column of the fornix bilaterally in MR images and found a volume of 78 ± 16 mm^3^ (mean ± SD) in the pre-HD group, 70 ± 18 mm^3^ in the symp-HD group and 118 ± 26 mm^3^ in the control group (Fig. 1g). We performed an ANOVA to analyze the fornix volume data between the three groups with sex, age and ICV as covariates. The statistical analyses demonstrated significant differences between the groups (*F*_1,85_ = 44.812, *p* < 0.0001) but no significant effect of sex, age or ICV on the fornix volume estimates (sex: *F*_1,85_ = 0.281, *p* = 0.597, age: *F*_1,85_ = 0.447, *p* = 0.506, ICV: *F*_1,85_ = 0.535, *p* = 0.466). Tukey’s post hoc analyses revealed significant differences between both HD groups and the control group (Fig. 1g) (control vs pre-HD, *p* < 0.0001; control vs symp-HD, *p* < 0.0001; pre-HD vs symp-HD, *p* = 0.289). As previous research has shown WM changes in the corpus callosum in HD [9, 45], we performed automated segmentation of the Image-HD cohort and found that there was a significant smaller volume of the corpus callosum in symp-HD compared to controls, but no significant difference between pre-HD and controls (Fig. 1h). We found no correlations between the estimated volume of the column of the fornix and ICV or hypothalamic volumes (Fig. 1, online resource; Spearman’s *ρ*, *p* = n.s.), indicating an early independent volumetric change in this structure.

We then wanted to determine whether there is any relationship between the fornix volume and clinical parameters in HD such as UHDRS-M, disease burden score, CAP scores, symptom duration, neurocognitive parameters (SDMT and STROOP) as well as neuropsychiatric parameters (FrSbe, SCOPI, HADS-A and HADS-B and BDI II). Correlation analyses were performed using Spearman’s *ρ* for the fornix volumes and the clinical, neurocognitive and neuropsychiatric data collected from all the participants. We found small but statistically significant correlations between fornix volume and age (*ρ*: − 0.230, *p* < 0.05), UHDRS (*ρ*, − 0.283, *p* < 0.05), SDMT (*ρ*: 0.414, *p* < 0.0001), STROOP (*ρ*: 0.419, *p* < 0.0001), HADS-A (Rh, *p* < 0.05), BDI II (*ρ*: − 0.392, *p* < 0.0001) as well as with symptom duration for the symp-HD group (*ρ*: − 0.371, *p* < 0.05) (Table 3, Fig. 1, online resource). There were no other significant correlations.

**Table 3:** Analyses of correlations between fornix and selected clinical, cognitive and neuropsychiatric data in the IMAGE-HD cohort

	Spearman’s *ρ*	*p* value
Age	− 0.230	0.027*
UHDRS-M	− 0.283	0.025*
CAG repeats	0.187	0.143
Disease burden score	0.037	0.774
Years to onset	− 0.295	0.107
CAP score	− 0.078	0.541
Symptom duration	− 0.371	0.037*
Verbal IQ	0.153	0.143
SDMT	0.414	< 0.0001***
Stroop	0.419	< 0.0001***
SCOPI	− 0.280	0.006**
FrSBE	− 0.140	0.180
HADS A	− 0.222	0.033*
HADS D	− 0.107	0.307
BDI II	− 0.392	< 0.0001***

*BDI II* Beck Depression Inventory Score Version II, *CAP* CAG age product, *FrSBe* Frontal Systems Behaviour Scale, *SCOPI* Schedule of Compulsions Obsessions and Pathological Impulses, *HADS A* Hospital Anxiety and Depression Scale—Anxiety Sub Score, *HADS D* Hospital Anxiety and Depression Scale—Depression Sub Score, *SDMT* Symbol Digit Modalities Test, *STROOP* STROOP speeded word reading task (number of correct words), *UHDRS-M* Unified Huntington’s Disease Rating Scale—Motor Subscale Score (Pre-HD, UHDRS < 5; Symp-HD, UHDRS ≥ 5)

Spearmans’ *ρ*, *p values < 0.05, **p values < 0.01, ***p values < 0.005

Taken together, the volume of the fornix is smaller in HD at an early stage before the clinical onset of motor symptoms compared to controls and the fornix volumes show discrete but statistically significant correlations to measures of depression as well as cognition.

### Smaller fornix volume in post-mortem HD tissue

**Fig. 2:**
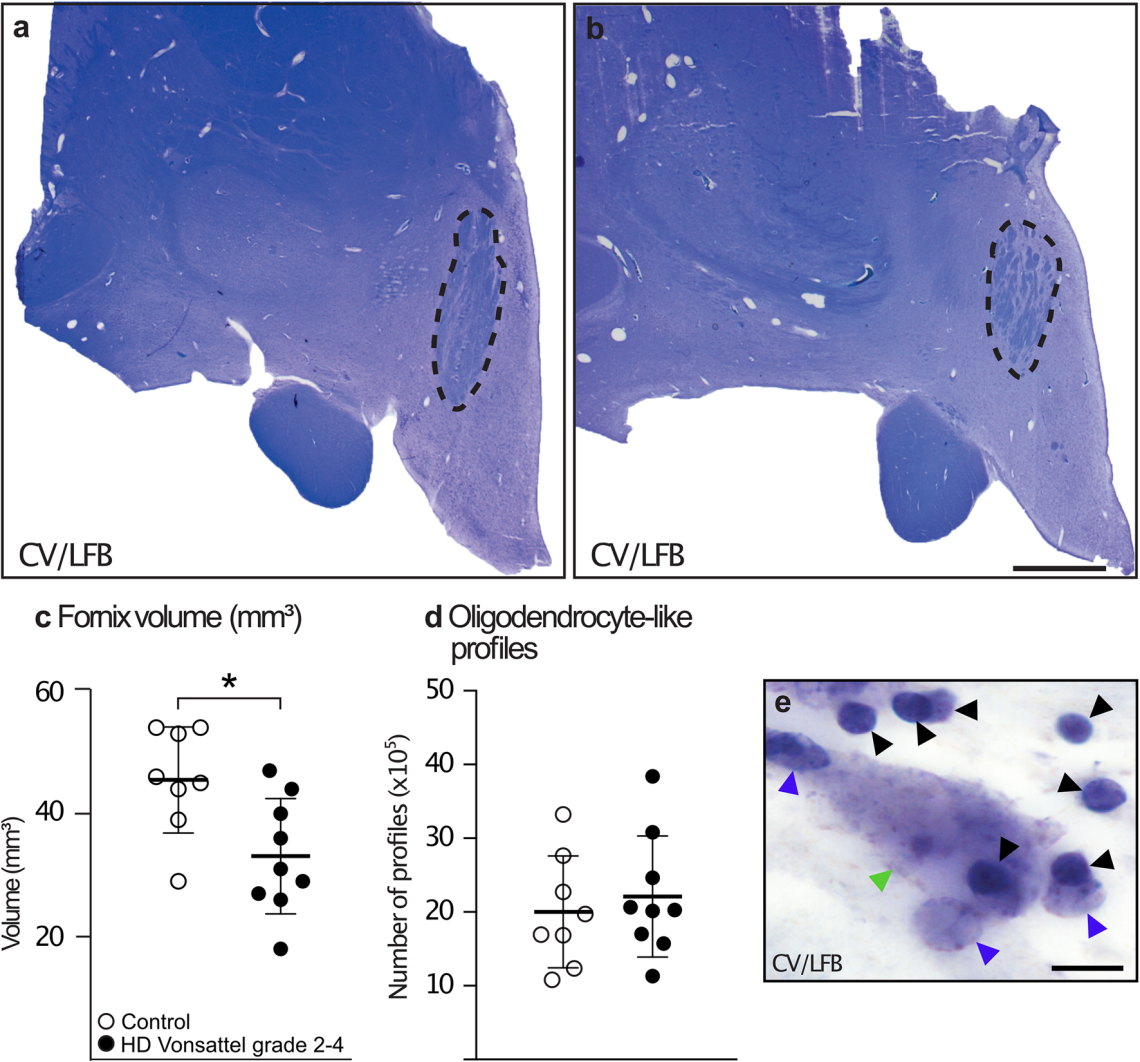
Smaller fornix volume with unaffected numbers of oligodendrocyte-like profiles in post-mortem tissue of HD cases. The volume of the fornix within the hypothalamic region was estimated in CV/LFB stained human post-mortem sections. Representative photographs of coronal sections of the hypothalamic regions with the dashed lines illustrating how the fornix region was delineated in a control case (**a**) and an HD case (**b**). The total volume of the fornix within the hypothalamic region was significantly smaller in the HD group (**c**). The total number of oligodendrocyte-like profiles assessed in the CV/LFB stained sections was similar between the groups (**d**). High-power photomicrographs of the CV/LFB stained sections in a control case (**e**) with black arrows pointing towards typical oligodendrocyte-like profiles, blue arrows pointing to glial-like profiles and green arrow pointing to a neuronal-like profile [19]. Data are expressed as mean ± SD. Student’s *t* test, *p < 0.05. Scale bar in **b**: 2000 μm; in **e**: 10 μm

We then used stereology to estimate the volume of the fornix within the hypothalamic region as well as the number of oligodendrocytes in 9 HD cases with Vonsattel grades 2–4 compared to 8 control cases (Fig. 2a, b, e). The stereological analyses were performed in 7 ± 1.6 sections in HD cases and in 7 ± 1.5 sections in control cases (Students *t* test, n.s.). We detected an average 27% smaller volume of the fornix in HD cases (Fig. 2c). Estimations of the total number of oligodendrocyte-like profiles within the studied fornix region revealed no changes in the total number of cells between HD and controls (Fig. 2d).

### Evidence of myelin breakdown in the fornix in HD

**Fig. 3:**
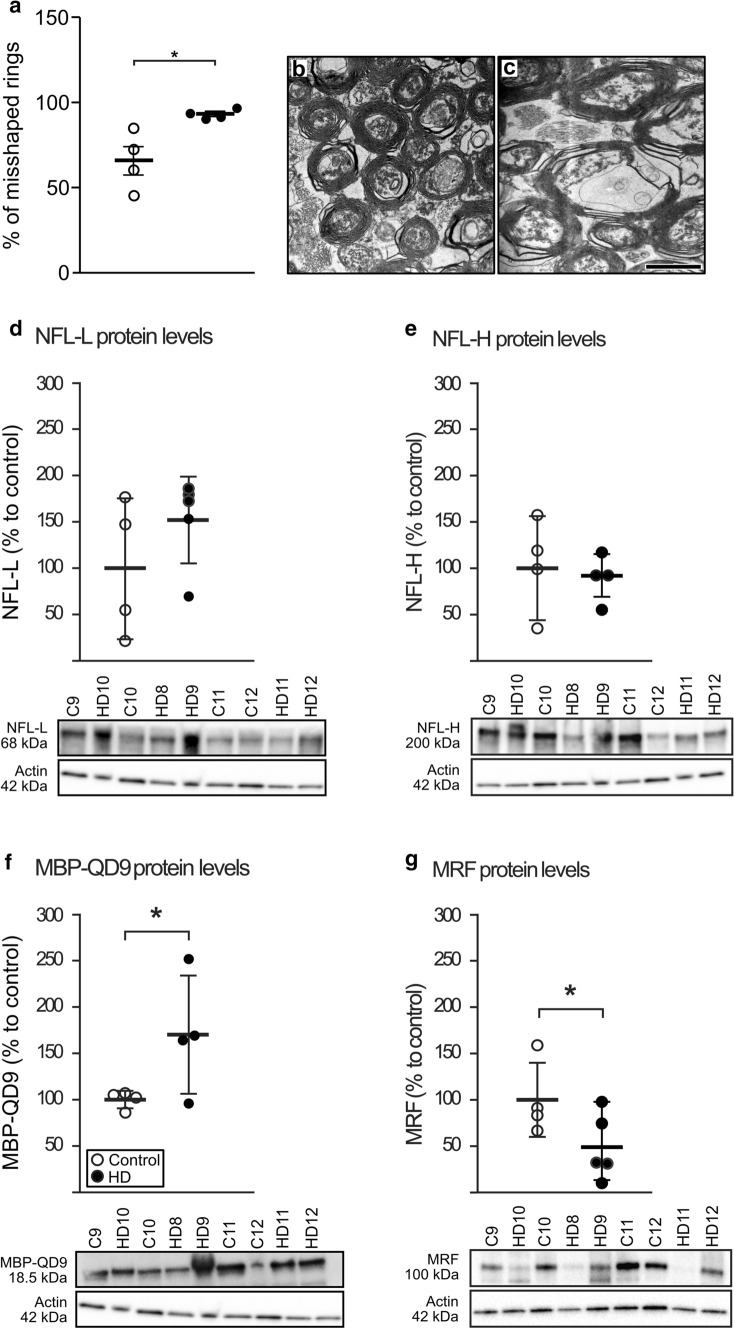
Evidence of myelin breakdown and reduced protein levels of MRF in the fornix in HD. The integrity of myelin rings in the fornix was evaluated on 46 randomly selected electron microscopy (EM) images from four HD and four healthy controls by a rater blinded to the identity of the cases. The number of misshapen myelin rings which contained sheets of swellings, disruptions, folds and irregular shape was significantly increased in HD cases compared to controls (**a**). Representative EM images from a control (**b**) and HD case (**c**). Western blot analysis of WM tissue of the fornix from five HD and four healthy controls revealed no significant differences in axonal markers, neurofilament light chain (**d**) and heavy chain (**e**), but a significant increase in MBP-QD9, a marker of demyelination (**f**) and significant decrease of myelin-regulating factor (MRF, **g**). The data are expressed as mean ± SD. Mann Whitney test, *p < 0.05. Scale bar in **c** 1000 nm

We then assessed the fornix at an ultrastructural level using EM in tissue from 4 HD cases compared to four controls. We detected a 27% increase in the percentage of misshapen myelin rings in HD cases compared to controls (Fig. 3a–c). There was no significant correlation between PMI and percentage of misshapen rings (Spearmans’ *ρ* 0,429, *p* = 0.289). In order to further investigate underlying mechanisms of the smaller fornix volume in HD, we wanted to examine whether there is axonal damage within this region. We examined protein levels of neurofilaments, which are neuron-specific cytoskeletal components present in axons. We found no significant differences in the protein levels of neurofilament light or heavy chain between HD and control cases (Fig. 3d, e), which indicates that axons per se in the fornix tract are intact in HD. We used the MBP-QD9 antibody, which has been shown to detect fragmented or damaged myelin [34, 35, 58], to further analyze the level of myelin breakdown in the fornix in HD. By Western blotting, we found an increase of 78% of MBP-QD9 in HD cases compared to controls (Fig. 3f; *n* = 4–5/group). Taken together, these data indicate myelin breakdown in the fornix in HD.

### Reduced expression of the myelin regulating factor (MRF) in the HD fornix

Next, we determined the protein levels of myelin regulating factor, MRF, a major transcription factor for oligodendrocyte genes, found to be reduced in mouse models of HD [24]. We found a reduction in MRF protein levels by 56% in HD cases compared to controls (Fig. 3g). This finding prompted us to profile the transcriptome of the fornix in HD.

### Transcriptome profiling reveals down-regulation of myelin and oligodendroglia genes in the HD fornix

**Fig. 4:**
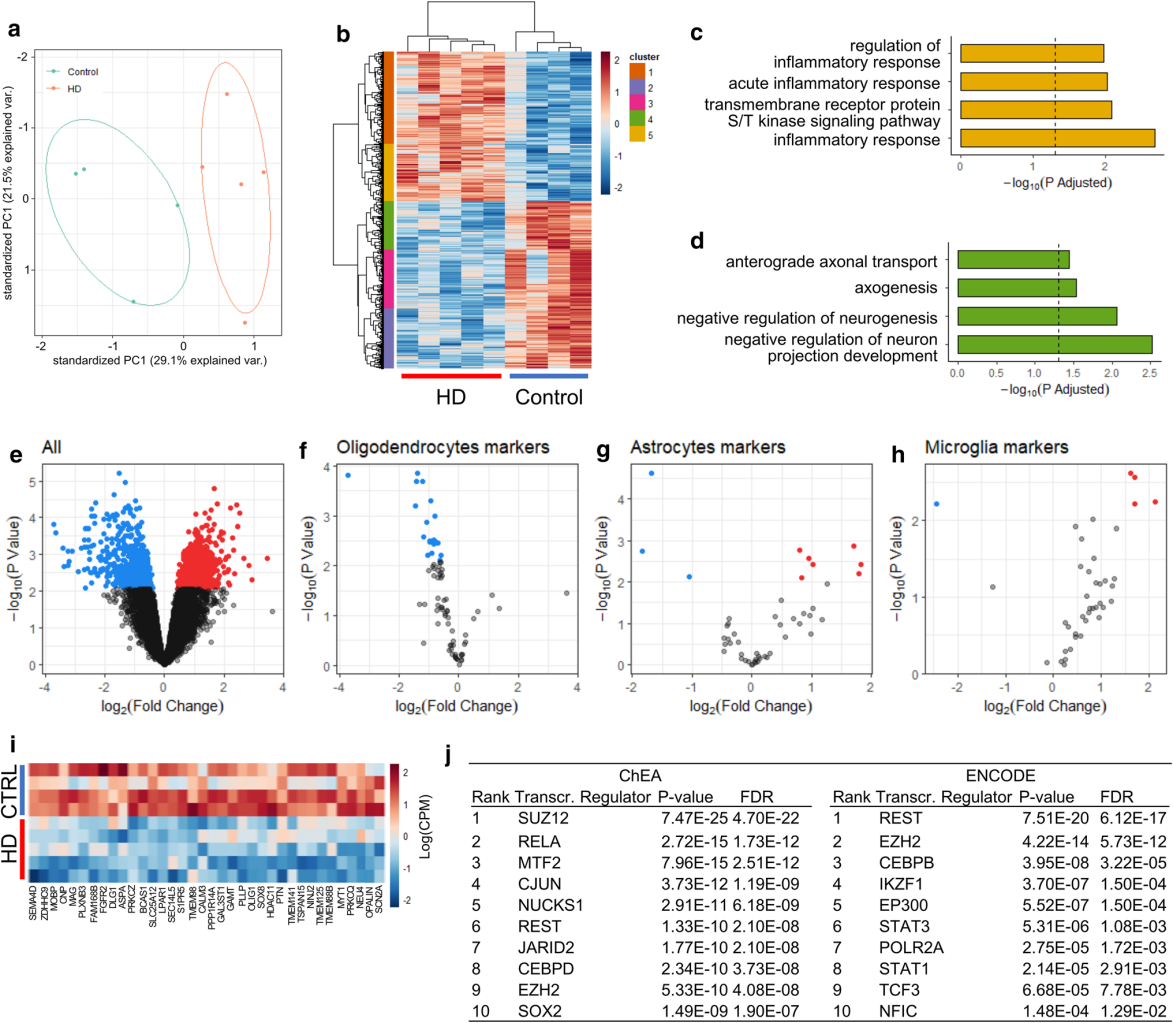
Transcriptome profiling reveals down-regulation of myelin and oligodendroglia genes in human HD fornix. RNA sequencing of WM tissue from the fornix of five HD cases and four healthy control cases. Principal component analysis (PCA) plot of control and HD fornix samples showing separation of experimental groups along the first principal component (PC1) (**a**). Heatmap of differentially expressed genes defines five separate subclusters of similarly regulated genes (**b**). Representative gene ontology terms related to subcluster 5 (yellow subcluster shown in **b**) comprised of genes with increased expression in HD fornix (**c**). Vertical dashed line is − log_10_(adjusted *p* value = 0.05). Representative gene ontology terms related to subcluster 4 shown in (green subcluster shown in **b**) comprised of genes with decreased expression in HD fornix (**d**). Vertical dashed line is − log_10_(adjusted *p* value = 0.05). Volcano plot showing all 1013 differentially expressed genes (FDR ≤ 0.10) (**e**). Volcano plot for oligodendroglia marker genes (**f**). Almost all oligodendrocyte-related genes are decreased in HD samples (log fold change < 0). Volcano plot for microglial marker genes. Almost all microglia-related genes are increased in HD samples (log fold change > 0) (**g**). Volcano plot for astrocytic marker genes (**h**). Heatmap of oligodendrocyte genes showing reduced expression in HD cases compared to control cases (measured as log CPM) (**i**). Transcription factor enrichment analysis shows that DEGs between HD and control cases are enriched for binding sites of SUZ12 and EZH2, components of PRC2, as well as REST (**j**)

To gain insight into the cellular and biological processes impacted in the fornix region in HD, we performed an unbiased, global transcriptome analysis using RNAseq on fornix samples from five HD cases and four control cases (Table 2). Principal component analysis (PCA) of log counts per million (CPM) values showed a clear separation of HD and control transcriptomes along the first principal component (PC) (Fig. 4a). Differential gene expression analysis found 507 genes with higher expression in HD samples, and 506 with lower (FDR ≤ 0.10, Fig. 4b, e) (Table 2, online resource). Subclustering and ontology analysis of genes with increased expression found terms related to immune activation (Fig. 4c). For genes with lower expression levels in HD samples, we found terms related to structural organisation of neuronal projections (Fig. 4d). To parse HD-associated glial changes, we focussed on glial cell markers identified in the human PanglaoDB single-cell database [17]. Virtually all microglia marker genes were increased in HD samples, while the majority of oligodendrocyte marker genes were decreased (Fig. 4e, f, g, h, i) (Table 3, online resource). This is likely to reflect down-regulation of the oligodendrocyte genes per cell as there was no difference in the number of oligodendrocytes in the fornix between HD and control cases.

To identify transcription factors and/or epigenetic mediators that may account for the differential gene expression patterns observed, we used EnrichR [28] to perform an analysis based on transcription factor/target-gene interactions using the ChEA and ENCODE databases of ChIP-based studies. The analysis revealed that differentially expressed genes (DEG) between HD and control cases are enriched for binding sites of SUZ12 and EZH2, components of the Polycomb Repressive Complex 2 (PRC2), as well as RE1 Regulation Transcription Factor (REST) (Fig. 4j).

## Discussion

**Fig. 5:**
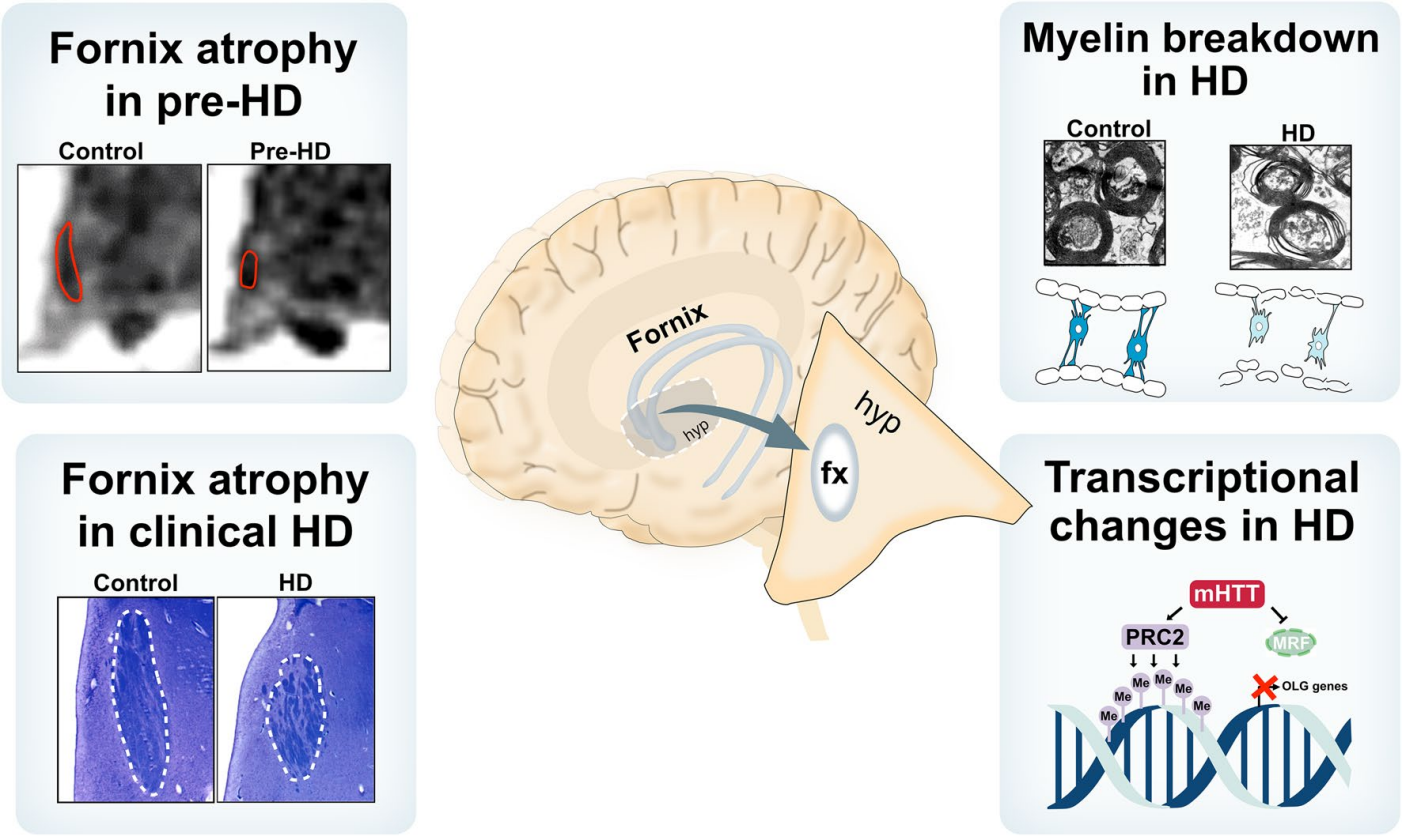
An overview of white matter changes in the fornix in HD. An illustration of white matter changes in the fornix identified in individuals with the *HD* gene in the present study

WM changes have emerged as important aspects of pathology in HD. Previous research has mainly focused on WM pathology in the corpus callosum and tracts related to the movement disorder. Psychiatric and cognitive symptoms are common in HD and are often present before motor disturbances. The biological origins of psychiatric symptoms in HD are not known but changes in the limbic system have been suggested to play a role (for review see [6]). In the present study we show that the volume of the fornix WM tract in the limbic system is smaller in prodromal HD using volumetric analyses of 3 T MRI on an individual level compared to controls. We further demonstrate that WM pathology includes a combination of myelin break-down and reduced transcription of oligodendrocyte genes. This may be associated with reduced levels of MRF, a key transcription factor for oligodendrocyte genes, as well as altered activity of PRC2 and REST. Taken together, our data indicate that myelin degradation and dysregulation of oligodendrocytes occur in the fornix in the limbic system in HD and that changes in this tract can be detected already in prodromal HD (summarized in Fig. 5).

The limbic system, including the fornix, is heavily involved in the regulation of emotion and cognition [31, 55]. This system has, however, not been extensively studied in HD [6]. An early study on seven HD cases showed loss of neurons in the entorhinal region and the subiculum, which are part of the limbic system with projections within the fornix [5]. One previous study has shown decreased fractional anisotropy in the fornix in a group of manifest HD using an automatic voxel-wide analyses based on multisubject diffusion tensor imaging data [8]. In the present study we show that volumetric analyses of the WM tract fornix within the hypothalamic region in both 3 T MRI and postmortem hypothalamic tissue reveal a smaller volume in HD. Interestingly, the smaller volume of the fornix is present already in prodromal HD indicating that WM loss occurs early in the disease process. Furthermore, we demonstrate statistically significant although small correlations between fornix volume and scores of depression, anxiety as well as cognitive measures, suggesting a link between psychiatric and cognitive symptoms and fornix pathology.

Oligodendrocytes and the myelin they generate are critical for axonal function. These cells are mainly generated during the postnatal period and differentiate from proliferative and migratory oligodendrocyte precursors (OPC). Myelination not only occurs in early life but is a plastic process in the adult brain important for learning, memory and as response to injury [60]. Dysfunction of OPCs has also been implicated in depressive disorder (for review see [63]). Here we show that the reduced fornix volume in HD is not due to a reduction in the number of oligodendrocyte-like profiles. Also, normal levels of neurofilament in this region suggest intact axons. These results argue against the possibility that the described loss of neurons in the entorhinal region and subiculum would play a role in the pathology found in the fornix. These results also render it unlikely that the reduced fornix volume is due to hypoplasia. Reduced brain growth and other neurodevelopmental aspects have been described in pre-manifest HD [30, 39]. Furthermore, low ABC scores in the HD group indicate that tauopathic and/or amyloid burden is not involved in the disease process. Instead, the reduced fornix volume appears to be due to reduced amount of myelin. Interestingly, degeneration of myelin and reduced myelin renewal has been suggested to play a role in age-related cognitive decline [57]. Here, we used the MBP-Q9 antibody that is raised against human myelin basic protein residues 69–88, an epitope only accessible in areas of myelin degeneration [34, 35]. We demonstrated increased levels of MBP-Q9 protein which indicates demyelination in the HD fornix [34, 35, 58]. This was further confirmed at the ultrastructural level using EM. Taken together, our data suggest degenerative changes of the myelin in HD.

Alterations in white matter and myelin may be due to direct effects of mutant HTT in oligodendrocytes in HD. This would also explain why there may be effects on myelin without apparent axonal damage. Reduced expression of a number of oligodendrocyte genes in the fornix of HD cases compared to controls as revealed by RNA sequencing is a key finding of this study. Analysis of differentially expressed genes based on transcription-factor/target-gene interactions revealed enrichment for binding sites of SUZ12 and EZH2, components of PRC2, as well as REST. These results are consistent with our recent findings in the BACHD mouse model of HD in which these genes were implicated in mutant HTT-mediated defects in oligodendroglia [14]. Abnormal REST activity has long been associated with neuronal pathology in HD, where it is thought to interfere with the expression of key trophic genes such as Brain Derived Neuroptrophic Factor [64]. However, the possible role of REST in oligodendroglial dysfunction is not well understood. PRC2, an epigenetic regulator mediating trimethylation of histone H3 lysine 27, has well-established roles in lineage determination and cell-type specification. In the context of oligodendroglia differentiation, down-regulation of PRC2 activity has been shown to parallel the maturation of oligodendrocytes [49].

We have shown that a number of molecular effects of PRC2 are increased in callosal tissues of BACHD mice and that inactivation of mutant HTT in oligodendroglia reverses these changes and rescues the demyelination and WM abnormalities in HD mice [14]. Our findings in the present study suggest that the contribution of dysregulated PRC2 activity to demyelination likely extends to human HD oligodendroglia and more widely influences WM regions beyond callosal tracts. These observations are consistent with studies showing a direct effect of mutant HTT on PRC2 activity and point to a broader role for PRC2 in HD [33, 47].

We also found reduced protein levels of MRF, a key transcriptional regulator that activates and maintains the expression of myelin genes in mature oligodendrocytes [12, 23]. MRF is only expressed in post-mitotic oligodendrocytes and not in OPCs. Previous studies in mice showed that mutant HTT abnormally binds to and mediates phosphorylation of MRF and thereby negatively affects its transcriptional activity [24, 61]. It has also been shown in mice that depletion of MRF prevents adult myelination reduces the generation of new oligodendrocytes in the adult brain and leads to reduced motor skill learning [11, 36]. Hence, the reduction in MRF protein is likely to be important for the downregulation of oligodendrocyte genes in WM in HD.

WM pathology has been demonstrated in several rodent models of HD including the YAC128 mouse and the BACHD mouse and rat models [14, 43, 53, 54]. The consistent findings of early WM pathology in both clinical HD and animal models of the disease have opened up the possibility for therapeutic interventions targeting WM in HD. In preclinical studies, laquinimod, an immunomodulatory agent, has been shown to rescue atrophy in the striatum, certain cortical regions as well as the corpus callosum in the YAC128 mouse [20]. Diffusion tensor imaging further showed that laquinimod improved microstructural abnormalities in the posterior corpus callosum (splenium) in HD mice [20]. Analysis of myelin ultrastructure in the posterior (splenium) region of the corpus callosum following laquinimod treatment demonstrated improvements in myelin sheath thickness and rescue of myelin constitutes such as Mbp and Plp1 [21]. These improvements in WM pathology were associated with improvements in several neurological and affective deficits including motor learning, motor function and depressive-like behaviour in YAC128 HD mice [20, 21]. Mechanistically, laquinimod was shown to improve myelination by influencing the post-translational modification of MRF in a manner that mitigated its inhibition by mutant HTT, thereby improving its myelin-promoting transcriptional activity [61]. These studies provide a proof-of-concept that improving myelination and WM deficits in HD pharmacologically may be possible using oligodendrocyte-targeted approaches, with potential to improve neurological outcomes in HD. Our data suggest that treatments for preventing demyelination in the fornix will need to occur early in the preclinical disease.

In conclusion, the present data show that the fornix in the limbic system is affected already at an early stage in the disease process in HD. The smaller fornix volume correlated with measures of psychiatric and cognitive changes, suggesting a role for demyelination of connections in the limbic system in these non-motor aspects of HD. Our data demonstrate in human post-mortem tissue that there is a significant downregulation of oligodendrocyte genes as well as signs of myelin breakdown in the fornix. Hence, demyelination of WM in HD is a significant part of the disease pathology and may, therefore, provide a promising target for therapeutic interventions.

## Supplementary Information

Below is the link to the electronic supplementary material.

Figure 1, online resource. Correlation analysis for fornix volume with cognitive and neuropsychiatric parameters. Overview of correlation analyses for fornix volume and selected clinical cognitive and neuropsychiatric data for all the participants in the IMAGE-HD cohort. No significant correlations were seen for ICV (a) and hypothalamic volume (b). Significant correlations were seen for age (c), UHDRS (d), SDMT (g), SCOPI (h), STROOP (i), HADS-A (j) and Beck Depression Inventory score Version II (j). Correlation analyses were made by Spearman’s rho tests. Abbreviations, HADS-A: Hospital Anxiety and Depression scale - anxiety sub score; SCOPI: Schedule of Compulsions Obsessions and Pathological Impulses; SDMT: Symbol Digit Modalities Test; STROOP, speeded word reading task (number of correct words); UHDRS-M: Unified Huntington’s Disease Rating Scale - motor subscale score (Pre-HD, UHDRS<5; Symp-HD, UHDRS≥5) (TIFF 2363 kb)

Supplementary file2 (DOCX 24 kb)

Supplementary file3 (XLSX 1136 kb)

Supplementary file4 (XLSX 26 kb)
