# Supplementary figures and images for: Early white matter pathology in the fornix of the limbic system in Huntington disease
Source: Acta Neuropathol. 2021 Aug 26;142(5):791–806. doi: 10.1007/s00401-021-02362-8 (PMC8500909; doi:10.1007/s00401-021-02362-8)

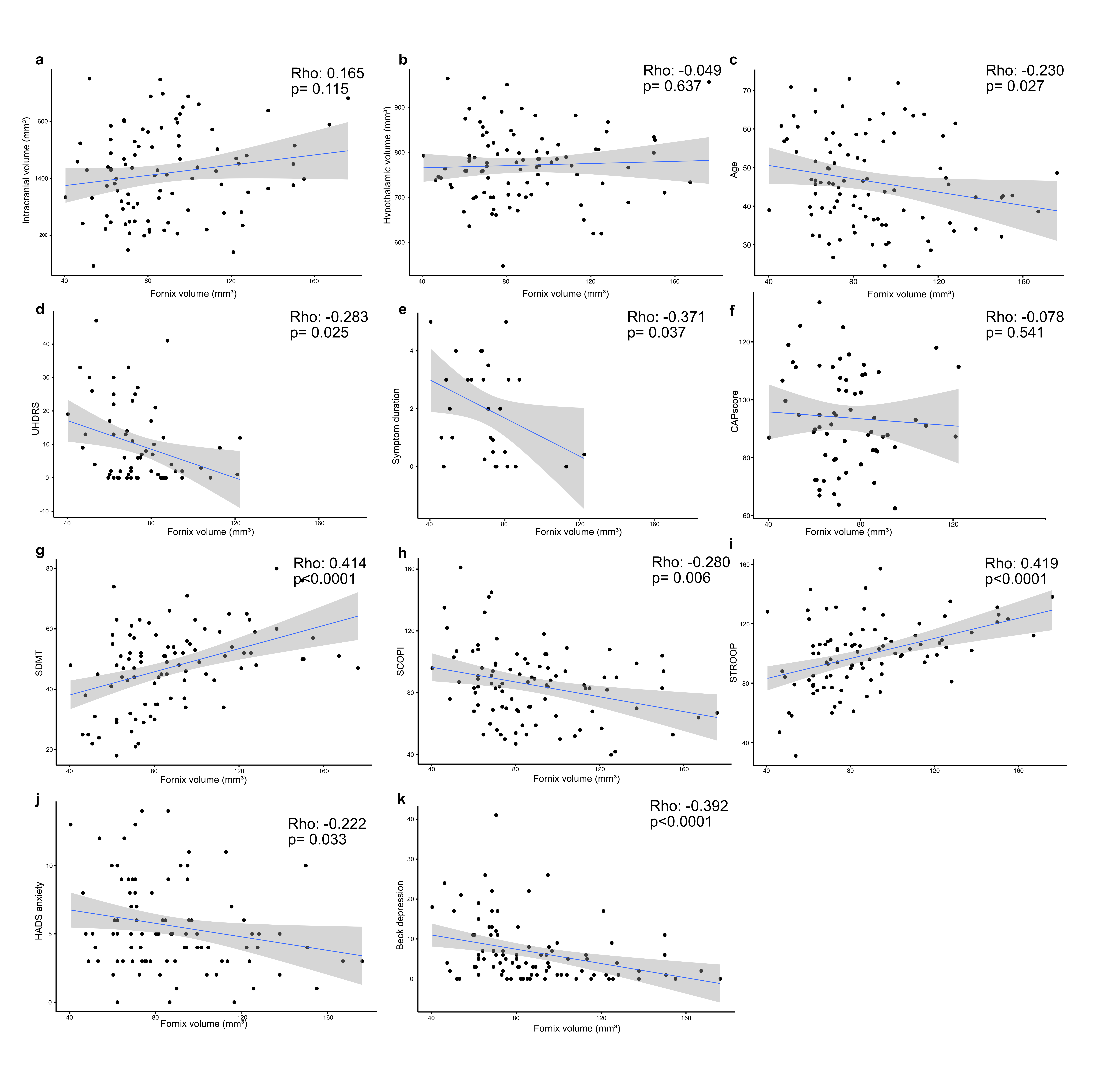

Supplement: Supplementary file 1 — Figure 1, online resource. Correlation analysis for fornix volume with cognitive and neuropsychiatric parameters. Overview of correlation analyses for fornix volume and selected clinical cognitive and neuropsychiatric data for all the participants in the IMAGE-HD cohort. No significant correlations were seen for ICV (a) and hypothalamic volume (b). Significant correlations were seen for age (c), UHDRS (d), SDMT (g), SCOPI (h), STROOP (i), HADS-A (j) and Beck Depression Inventory score Version II (j). Correlation analyses were made by Spearman’s rho tests. Abbreviations, HADS-A: Hospital Anxiety and Depression scale - anxiety sub score; SCOPI: Schedule of Compulsions Obsessions and Pathological Impulses; SDMT: Symbol Digit Modalities Test; STROOP, speeded word reading task (number of correct words); UHDRS-M: Unified Huntington’s Disease Rating Scale - motor subscale score (Pre-HD, UHDRS<5; Symp-HD, UHDRS≥5) (TIFF 2363 kb) [file 401_2021_2362_MOESM1_ESM.tiff]
