# Supplementary material for: Early white matter pathology in the fornix of the limbic system in Huntington disease
Source: Acta Neuropathol. 2021 Aug 26;142(5):791–806. doi: 10.1007/s00401-021-02362-8 (PMC8500909; doi:10.1007/s00401-021-02362-8)
Supplement: Supplementary file 2 — Supplementary file2 (DOCX 24 kb) [file 401_2021_2362_MOESM2_ESM.docx]

**Early white matter pathology in the fornix of the limbic system in Huntington disease**.

Original article to Acta Neuropathologica

Authors: Sanaz Gabery, Jing Eugene Kwa, Rachel Y Cheong, Barbara Baldo, Costanza Ferrari Bardile, Brendan Tan, Catriona McLean, Nellie Georgiou-Karistiani^4^, Govinda R Poudel, Glenda Halliday, Mahmoud A. Pouladi, Åsa Petersén^.^ CA: Åsa Petersén: Translational Neuroendocrine Research Unit, Department of Experimental Medical Science, Lund University, Lund, Sweden. E-mail: asa.petersen@med.lu.se

**Table 1, online resource. Overview of glial markers.** The list of markers was derived from human single-cell RNA-sequencing data (PanglaoDB, [17]).

| Oligodendrocytes | Astrocytes | Microglia |
| --- | --- | --- |
| GALC | S100B | ITGAM |
| MOG | SLC1A2 | ITGAX |
| OLIG1 | GFAP | P2RY12 |
| OLIG2 | BYSL | P2RX7 |
| TF | ALDH1L1 | TREM2 |
| SOX10 | SLC1A3 | TMEM119 |
| GJC2 | FGFR3 | SALL1 |
| GAMT | AQP4 | CSF3R |
| BCAS1 | HTRA1 | TCIRG1 |
| ENPP6 | CLDN10 | SLC2A5 |
| TMEM63A | NKAIN4 | IL10RA |
| GPR62 | ACSL6 | ENTPD1 |
| BACE1 | ACSBG1 | TMEM173 |
| CREB5 | TRIL | TBXAS1 |
| FGFR2 | AGT | CEBPA |
| SLC25A38 | GPR37L1 | OLFML3 |
| HDAC11 | SLC4A4 | TGFBR1 |
| TYRO3 | NTSR2 | LPCAT2 |
| S1PR5 | SLC6A11 | ITGB5 |
| ASPA | HSPA2 | COLEC12 |
| EPHB1 | SLC16A2 | AEBP1 |
| HEPACAM | SYNE1 | ADAP2 |
| SOX8 | DIO2 | SLCO2B1 |
| FASN | ENTPD2 | PTGS1 |
| OMG | HSPB6 | APBB1IP |
| ZDHHC9 | PLXNB1 | MERTK |
| ADAMTS4 | LCAT | PLXDC2 |
| MYRF | CMTM5 | MAFB |
| SEMA4D | S1PR1 | CMTM6 |
| TMEM125 | LUZP2 | UCP2 |
| KLHL2 | GSTA4 | TNFRSF1B |
| DPY19L1 | SLC25A28 | CLEC3B |
| TSPAN15 | SLC25A46 | CD53 |
| HAPLN2 | TEAD1 | FOS |
| NKX6-2 | ALDOC | EGR1 |
| GJB1 | GJA1 | AIF1 |
| OPALIN | APOE | CTSS |
| VLDLR | SOX9 | CSF1R |
| TMEM163 | VIM | SKI |
| ERMN | FABP7 | C1QB |
| FA2H | SOCS3 |  |
| CLDN11 | SYNM |  |
| ANLN | HMG20A |  |
| ELOVL7 | NFATC3 |  |
| PLLP | SRR |  |
| NINJ2 | ETS1 |  |
| SGK2 | SLIT1 |  |
| GPR37 | SNPH |  |
| IL33 |  |  |
| KLK6 |  |  |
| NFE2L3 |  |  |
| PEX5L |  |  |
| ITGB4 |  |  |
| MYO1D |  |  |
| PDE8A |  |  |
| CNTF |  |  |
| EML1 |  |  |
| KIF13B |  |  |
| KIF6 |  |  |
| NIPAL4 |  |  |
| SEC14L5 |  |  |
| ST18 |  |  |
| DUSP15 |  |  |
| PRKCZ |  |  |
| NPC1 |  |  |
| MAG |  |  |
| SLC25A29 |  |  |
| SLC25A19 |  |  |
| PLEKHH1 |  |  |
| TMEM88B |  |  |
| DBNDD2 |  |  |
| EFNB3 |  |  |
| SGK3 |  |  |
| PNPLA2 |  |  |
| ARRDC2 |  |  |
| ENPP2 |  |  |
| PLP1 |  |  |
| ITPR2 |  |  |
| MBP |  |  |

Abbreviations: ACSBG1; Acyl-CoA Synthetase Bubblegum Family Member 1, ACSL6; Acyl-CoA Synthetase Long Chain Family Member 6, ADAMTS4; ADAM Metallopeptidase With Thrombospondin Type 1 Motif 4, ADAP2; ArfGAP With Dual PH Domains 2, AEBP1; AE Binding Protein 1, AGT; Angiotensinogen, AIF1; Allograft Inflammatory Factor 1, ALDH1L1; Aldehyde Dehydrogenase 1 Family Member L1, ALDOC; Aldolase, Fructose-Bisphosphate C, ANLN; Anillin Actin Binding Protein, APBB1IP; Amyloid Beta Precursor Protein Binding Family B Member 1 Interacting Protein, APOE; Apolipoprotein E, AQP4; Aquaporin 4, ARRDC2; Arrestin Domain Containing 2, ASPA; Aspartoacylase, BACE1; Beta-Secretase 1, BCAS1; Brain Enriched Myelin Associated Protein 1, BYSL; Bystin Like, C1QB; Complement C1q B Chain, CD53; CD53 Molecule, CEBPA; CCAAT Enhancer Binding Protein Alpha, CLDN10; Claudin 10, CLDN11; Claudin 11, CLEC3B; C-Type Lectin Domain Family 3 Member B, CMTM5; CKLF Like MARVEL Transmembrane Domain Containing 5, CMTM6; CKLF Like MARVEL Transmembrane Domain Containing 6, CNTF; Ciliary Neurotrophic Factor, COLEC12; Collectin Subfamily Member 12, CREB5; CAMP Responsive Element Binding Protein 5, CSF1R; Colony Stimulating Factor 1 Receptor, CSF3R; Colony Stimulating Factor 3 Receptor, CTSS; Cathepsin S, DBNDD2; Dysbindin Domain Containing 2, DIO2; Iodothyronine Deiodinase 2, DPY19L1; Dpy-19 Like C-Mannosyltransferase 1, DUSP15; Dual Specificity Phosphatase 15, EFNB3; Ephrin B3, EGR1; Early Growth Response 1, ELOVL7; ELOVL Fatty Acid Elongase 7, EML1; EMAP Like 1, ENPP2; ENPP2, ENPP6; Ectonucleotide Pyrophosphatase/Phosphodiesterase 6, ENTPD1; Ectonucleoside Triphosphate Diphosphohydrolase 1, ENTPD2; Ectonucleoside Triphosphate Diphosphohydrolase 2, EPHB1; EPH Receptor B1, ERMN; Ermin, ETS1; ETS Proto-Oncogene 1, Transcription Factor,

FABP7; Fatty Acid Binding Protein 7, FASN; Fatty Acid Synthase, FGFR2; Fibroblast Growth Factor Receptor 2, FGFR3; Fibroblast Growth Factor Receptor 3, FOS; Fos Proto-Oncogene, AP-1 Transcription Factor Subunit, GALC; Galactosylceramidase, GAMT; Guanidinoacetate N-Methyltransferase, GJA1; Gap Junction Protein Alpha 1, GJB1; Gap Junction Protein Beta 1, GJC2; Gap Junction Protein Gamma 2, GPR37; G Protein-Coupled Receptor 37, GPR37L1; G Protein-Coupled Receptor 37 Like 1, GPR62; G Protein-Coupled Receptor 62, HAPLN2; Hyaluronan And Proteoglycan Link Protein 2, HDAC11; Histone Deacetylase 11, HEPACAM; Hepatic And Glial Cell Adhesion Molecule, HMG20A; High Mobility Group 20A, HSPA2; Heat Shock Protein Family A (Hsp70) Member 2, HSPB6; Heat Shock Protein Family B (Small) Member 6, HTRA1; HtrA Serine Peptidase 1, IL10RA; Interleukin 10 Receptor Subunit Alpha, IL33; Interleukin 33, ITGAM; Integrin Subunit Alpha M, ITGAX; Integrin Subunit Alpha X, ITGB4; Integrin Subunit Beta 4, ITGB5; Integrin Subunit Beta 5, ITPR2; Inositol 1,4,5-Trisphosphate Receptor Type 2, KIF13B; Kinesin Family Member 13B, KIF6; Kinesin Family Member 6, KLHL2; Kelch Like Family Member 2, KLK6; Kallikrein Related Peptidase 6, LCAT; Lecithin-Cholesterol Acyltransferase, LPCAT2; Lysophosphatidylcholine Acyltransferase 2, LUZP2; Leucine Zipper Protein 2, MAFB; MAF BZIP Transcription Factor B, MAG; Myelin Associated Glycoprotein, MBP; Myelin Basic Protein, MERTK; MER Proto-Oncogene, Tyrosine Kinase, MOG; Myelin Oligodendrocyte Glycoprotein, MYO1D; Myosin ID, MYRF; Myelin Regulatory Factor, NFATC3; Nuclear Factor Of Activated T Cells 3, NFE2L3; Nuclear Factor, Erythroid 2 Like 3, NINJ2; Ninjurin 2, NIPAL4; NIPA Like Domain Containing 4, NKAIN4; Sodium/Potassium Transporting ATPase Interacting 4, NKX6-2; NK6 Homeobox 2, NPC1; NPC Intracellular Cholesterol Transporter 1, NTSR2; Neurotensin Receptor 2, OLFML3; Olfactomedin Like 3, OLIG1; Oligodendrocyte Transcription Factor 1, OLIG2; Oligodendrocyte Transcription Factor 2, OMG; Oligodendrocyte Myelin Glycoprotein, OPALIN; Oligodendrocytic Myelin Paranodal And Inner Loop Protein, P2RX7; Purinergic Receptor P2X 7, P2RY12; Purinergic Receptor P2Y12, PDE8A; Phosphodiesterase 8A, PEX5L; Peroxisomal Biogenesis Factor 5 Like, PLEKHH1; Pleckstrin Homology, MyTH4 And FERM Domain Containing H1, PLLP; Plasmolipin, PLP1; Proteolipid Protein 1, PLXDC2; Plexin Domain Containing 2, PLXNB1; Plexin B1, PNPLA2; Patatin Like Phospholipase Domain Containing 2, PRKCZ; Protein Kinase C Zeta, PTGS1; Prostaglandin-Endoperoxide Synthase 1, S100B; S100 Calcium Binding Protein B, S1PR1; Sphingosine-1-Phosphate Receptor 1, S1PR5; Sphingosine-1-Phosphate Receptor 5, SALL1; Spalt Like Transcription Factor 1, SEC14L5; SEC14 Like Lipid Binding 5, SEMA4D; Semaphorin 4D, SGK2; Serum/Glucocorticoid Regulated Kinase 2, SGK3; Serum/Glucocorticoid Regulated Kinase Family Member 3, SKI; SKI Proto-Oncogene, SLC16A2; Solute Carrier Family 16 Member 2, SLC1A2; Solute Carrier Family 1 Member 2, SLC1A3; Solute Carrier Family 1 Member 3, SLC25A19; Solute Carrier Family 25 Member 19, SLC25A28; Solute Carrier Family 25 Member 28, SLC25A29; Solute Carrier Family 25 Member 29, SLC25A38; Solute Carrier Family 25 Member 38, SLC25A46; Solute Carrier Family 25 Member 46, SLC2A5; Solute Carrier Family 2 Member 5, SLC4A4; Solute Carrier Family 4 Member 4, SLC6A11; Solute Carrier Family 6 Member 11, SLCO2B1; Solute Carrier Organic Anion Transporter Family Member 2B1, SLIT1; Slit Guidance Ligand 1, SNPH; Syntaphilin, SOCS3; Suppressor Of Cytokine Signaling 3, SOX10; SRY-Box Transcription Factor 10, SOX8; SRY-Box Transcription Factor 8, SOX9; SRY-Box Transcription Factor 9, SRR; Serine Racemase, ST18; C2H2C-Type Zinc Finger Transcription Factor, SYNE1; Spectrin Repeat Containing Nuclear Envelope Protein 1, SYNM; Synemin, TBXAS1; Thromboxane A Synthase 1, TCIRG1; T Cell Immune Regulator 1, ATPase H+ Transporting V0 Subunit A3, TEAD1; TEA Domain Transcription Factor 1, TF; Transferrin, TGFBR1; Transforming Growth Factor Beta Receptor 1, TMEM119; Transmembrane Protein 119, TMEM125; Transmembrane Protein 125, TMEM163; Transmembrane Protein 163, TMEM173; Stimulator Of Interferon Response CGAMP Interactor 1, TMEM63A; Transmembrane Protein 63A, TMEM88B; Transmembrane Protein 88B, TNFRSF1B; TNF Receptor Superfamily Member 1B, TREM2; Triggering Receptor Expressed On Myeloid Cells 2, TRIL; TLR4 Interactor With Leucine Rich Repeats, TSPAN15; Tetraspanin 15, TYRO3; Protein Tyrosine Kinase, UCP2; Uncoupling Protein 2, VIM; Vimentin, VLDLR; Very Low Density Lipoprotein Receptor, ZDHHC9; Zinc Finger DHHC-Type Palmitoyltransferase 9.
